# Supplementary material for: Validation of Aerobic Capacity (VO2max) and Pulse Oximetry in Wearable Technology
Source: Sensors (Basel). 2025 Jan 6;25(1):275. doi: 10.3390/s25010275 (PMC11723475; doi:10.3390/s25010275)
Supplement: Supplementary file 1 [file sensors-25-00275-s001.zip › sensors-3344344-supplementary.pdf]

**Supplementary Table S1.** Validity statistics by condition.

| | Fenix Anterior Normoxia | Criterion Anterior Normoxia | Fenix Posterior Normoxia | Criterion Posterior Normoxia | Fenix Anterior Hypoxia | Criterion Anterior Hypoxia | Fenix Posterior Hypoxia | Criterion Posterior Hypoxia |
|---|---|---|---|---|---|---|---|---|
| Mean (%) | 95.67% | 97.10% | 95.74% | 97.05% | 94.90% | 88.00% | 95.18% | 85.90% |
| Standard Deviation | 2.35% | 1.74% | 1.41% | 2.24% | 0.32% | 8.29% | 1.66% | 9.55% |
| MAPE | | 1.80% | | 2.95% | | 6.93% | | 6.92% |
| Pearson Correlation | | 0.44 | | -0.47 | | 0.00 | | 0.29 |
| Lin's Concordance | | 0.37 | | -0.33 | | 0.00 | | 0.10 |
| Bland-Altman Bias | | -1.08 (-2.53, 0.36) | | -1.37 (-2.95, 0.22) | | 4.90 (0.56, 9.24) | | 4.36 (0.30, 8.43) |
| TOST Test (Upper) | | 0.003 | | < 0.001 | | 0.82 | | 0.76 |
| TOST Test (Lower) | | 0.47 | | 0.36 | | 0.001 | | 0.001 |
